# A genomic and transcriptomic approach for a differential diagnosis between primary and secondary ovarian carcinomas in patients with a previous history of breast cancer

**DOI:** 10.1186/1471-2407-10-222

**Published:** 2010-05-21

**Authors:** Jean-Philippe Meyniel, Paul H Cottu, Charles Decraene, Marc-Henri Stern, Jérôme Couturier, Ingrid Lebigot, André Nicolas, Nina Weber, Virginie Fourchotte, Séverine Alran, Audrey Rapinat, David Gentien, Sergio Roman-Roman, Laurent Mignot, Xavier Sastre-Garau

**Affiliations:** 1 Institut Curie, Department of Translational Research, 26 rue d'Ulm, 75248 Paris, Cedex 05, France; 2 Institut Curie, Department of Medical Oncology, 26 rue d'Ulm, 75248 Paris, Cedex 05, France; 3 Institut Curie, Research Unit, 26 rue d'Ulm, 75248 Paris, Cedex 05, France; 4 CNRS, UMR144, 26 rue d'Ulm, 75248 Paris, Cedex 05, France; 5 INSERM, U830, 26 rue d'Ulm, 75248 Paris, Cedex 05, France; 6 Institut Curie, Department of Pathology, 26 rue d'Ulm, 75248 Paris, Cedex 05, France; 7 Institut Curie, Department of Surgery, 26 rue d'Ulm, 75248 Paris, Cedex 05, France

## Abstract

**Background:**

The distinction between primary and secondary ovarian tumors may be challenging for pathologists. The purpose of the present work was to develop genomic and transcriptomic tools to further refine the pathological diagnosis of ovarian tumors after a previous history of breast cancer.

**Methods:**

Sixteen paired breast-ovary tumors from patients with a former diagnosis of breast cancer were collected. The genomic profiles of paired tumors were analyzed using the Affymetrix GeneChip^® ^Mapping 50 K Xba Array or Genome-Wide Human SNP Array 6.0 (for one pair), and the data were normalized with ITALICS (ITerative and Alternative normaLIzation and Copy number calling for affymetrix Snp arrays) algorithm or Partek Genomic Suite, respectively. The transcriptome of paired samples was analyzed using Affymetrix GeneChip^® ^Human Genome U133 Plus 2.0 Arrays, and the data were normalized with gc-Robust Multi-array Average (gcRMA) algorithm. A hierarchical clustering of these samples was performed, combined with a dataset of well-identified primary and secondary ovarian tumors.

**Results:**

In 12 of the 16 paired tumors analyzed, the comparison of genomic profiles confirmed the pathological diagnosis of primary ovarian tumor (n = 5) or metastasis of breast cancer (n = 7). Among four cases with uncertain pathological diagnosis, genomic profiles were clearly distinct between the ovarian and breast tumors in two pairs, thus indicating primary ovarian carcinomas, and showed common patterns in the two others, indicating metastases from breast cancer. In all pairs, the result of the transcriptomic analysis was concordant with that of the genomic analysis.

**Conclusions:**

In patients with ovarian carcinoma and a previous history of breast cancer, SNP array analysis can be used to distinguish primary and secondary ovarian tumors. Transcriptomic analysis may be used when primary breast tissue specimen is not available.

## Background

Malignant ovarian tumors comprise a wide and heterogeneous collection of primary and secondary tumors. In patients who had previously developed a breast cancer, the differential diagnosis between primary ovarian carcinoma and metastases from breast cancer may be sometimes challenging, while it is mandatory to ensure the optimal care for patients. Indeed, metastatic spread and biological pattern differ between metastatic breast cancer and primary ovarian carcinoma. Metastatic breast cancer assessment requires whole body CT-scan, bone scan, and CA 15-3 measurement. Primary ovarian carcinoma assessment requires extensive intra-abdominal exploration and CA-125 measurement. Moreover, the prognosis of metastatic breast cancer and primary ovarian carcinoma widely differs, with a median progression-free survival ranging from 20 to 40 months, and from 9 to 30 months, respectively [1,2]. Most importantly, therapeutic options are very different. Usual therapies for advanced breast cancer may combine or alternate hormonotherapy, chemotherapy regimens, and targeted therapies according to the tumor profile. Conversely, medical therapeutic options for ovarian cancer are scarce, based on paclitaxel-carboplatin combination. Other drugs have been shown to provide minor benefits to the patients, and targeted therapies are only entering early clinical trials.

Surgery is required to provide a thorough exploration of the abdominal cavity, to remove malignant ovarian lesions, to obtain a diagnosis, which is crucial for prognosis, and to plan adequate treatment [3]. In surgical series, ovarian metastases from other primary cancers represent 5% to 20% of all ovarian cancers [3-5]. Metastatic lesions to the ovaries are more commonly seen from primary colon cancer, appendiceal, and breast carcinomas. However, there are few clinical or pathological features that make possible to arrive at a differential diagnosis between primary and secondary tumors [6,7].

BRCA1 and BRCA2 mutation carriers have an increased risk of primary breast and ovarian tumors [8], whereas patients with an infiltrating lobular carcinoma (ILC) of the breast are more likely to develop secondary ovarian metastases [9,10]. To date, pathological examination remains the cornerstone of the differential diagnosis between primary ovarian tumor and ovarian metastases. In case of metastatic lesions, the involvement of ovaries is more often bilateral, and associated with ascites [3-5]. In those tumors, the pathological feature is more often a stromal rather than a serous invasion, suggesting a metastatic diffusion through blood and lymphatic vessels [5]. In that case, the differential diagnosis is of paramount importance. Several approaches have been developed to discriminate between primary and secondary ovarian cancers. Among them, immunohistochemistry (IHC) has evaluated diagnostic markers, presumably able to support the diagnosis, such as PAX8, a transcription factor for organogenesis of Müllerian system, or Wilms tumor suppressor gene (WT1) whose expression is regulated by PAX8 [11,12]. However, only limited series, without validation data, have been reported so far. We speculate that genomic analysis may represent an alternative approach, which could also add some robustness to PAX8 IHC data. To our knowledge, only one study has evaluated high throughput genomic analyses to refine the diagnosis of ovarian lesions following an adenocarcinoma of the endometrium [13]. In a series of nine patients, using 19 k comparative genomic hybridization (CGH) arrays, the authors established that, in three cases out of nine for which the pathological diagnosis was equivocal, the genomic analyses were able to provide a clear diagnosis. In this series, no transcriptomic data were reported.

In the present work, we proposed to develop genomic and transcriptomic approaches to discriminate between primary and secondary ovarian tumors after breast cancer when the pathological diagnosis was equivocal. For this purpose, we characterized genomic and transcriptomic profiles in paired breast and ovarian tumors diagnosed in a same patient, and compared genomic data with clinical and pathological characteristics.

## Methods

### Clinical cases and data

Sixteen patients who had developed an infiltrating breast carcinoma and a subsequent ovarian tumor, and for whom frozen tissues were available for both tumors were included in the study. Breast tumors were classified according to the American Joint Committee on Cancer (AJCC)/Union Internationale Contre le Cancer (UICC) staging system and usual pathological parameters [14]. Ovarian tumors were classified according to the Fédération Internationale de Gynécologie Obstétrique (FIGO) staging system.

Information about surgical and systemic treatments, and the occurrence of other metastatic sites were collected. The clinical data were reviewed by two medical oncologists (PHC, LM). Pathological data were blindly reviewed by two pathologists (NW, XSG), and, in case of discrepancy, a consensus was achieved. To ensure an independent data extraction, all procedures were conducted separately by reviewers, and samples were analyzed without knowledge of their supposed status (*i.e*. primary tumor, or metastasis).

This study was approved by the Institutional Review Board and Ethics committee. Patients were informed that their biological samples could be used for research purposes and that they had the right to refuse if they so wished.

### Immunohistochemical analysis

Immunohistochemical analysis was performed on formalin-fixed paraffin-embedded tissue sections (depth: 3 μm). The samples were deparaffinized and pretreated in EDTA buffer at pH 9 (40 minutes at 97°C), and then hydrated in PBS solution for 5 minutes. Then, the rabbit polyclonal anti-PAX8 antibody (Protein Tech Group Inc., Chicago, IL, USA) was applied (dilution: 1/200), and samples were incubated overnight at 4°C. The endogenous peroxidase activity was blocked with hydrogen peroxide. A second antibody directed against the primary anti-PAX8 antibody and coupled with a peroxidase polymer Envision+ (Dako, Trappes, France) was applied for 30 minutes. Then, the peroxidase was revealed during a 10-minute incubation with a di-aminobenzidine solution (DAB Dako K3468). Finally, samples were counterstained with haematoxylin (2 minutes), and mounted with permanent media.

### DNA and RNA extraction and preparation for microarray experiment

Tumor DNA and RNA were provided by the Biological Resource Center of the Institut Curie. Prior to DNA and RNA isolation, a tissue section of tumor fragments was performed and stained with hematoxylin and eosin to evaluate tumor cellularity. All analyzed tumors had more than 50% of tumoral cells on the tissue section. The DNA was extracted from frozen tumor samples using a standard phenol/chloroform procedure. The total RNA was isolated using TRIzol reagent (Invitrogen, Cergy-Pontoise, France) in accordance with the manufacturers' instructions. The concentration of RNA was measured by absorbance at 260 nm. The quality of each RNA sample was determined with Agilent 2100 bioanalyzer. RNAs were processed on chips only if the following criteria were fulfilled: RIN (a measure of RNA quality) ≥ 7.6, (28S/18S) ≥ 1.8, (260 nm/230 nm) ≥ 1.8, and (260 nm/280 nm) ≥ 1.8. Targets were prepared according to Affymetrix (Affymetrix Inc., Santa Clara, CA, USA) One Cycle Synthesis protocol, starting from 2 μg of total RNA. Targets were hybridized to GeneChip^® ^Human Genome U133 plus 2.0 Arrays if yield and size of targets were reached. Twenty micrograms of complementary RNA, with a specific size distribution were used to hybridize GeneChip^® ^Human Genome U133 plus 2.0 Array.

Regarding DNA, the quality was assessed on agarose gel, if a smear was observed instead of a band, the sample was discarded. A 250-ng genomic DNA was used to generate targets according to the GeneChip^® ^Mapping 50 K Xba protocol or Genome-Wide Human SNP Array 6.0 protocol. Targets were prepared if 45 μg of amplified DNA were available and if their size was between 250 and 2,000 bp, and hybridized according to manufacturer's recommendations.

### 50 k SNP Array and SNP6.0 Array data analysis

Intensity signals data from Genechip^® ^Human Mapping 50 K Xba Array or Genome-Wide Human SNP Array 6.0 were normalized and analyzed using ITALICS (ITerative and Alternative normaLIzation and Copy number calling for affymetrix Single nucleotide polymorphism [SNP] arrays) algorithm [15] or Partek Genomic Suite (Partek Inc., St Louis, MO, USA), respectively. The detection and determination of genomics events (gains, losses, amplifications and breakpoints) was performed using GLAD (Gain and Loss Analysis of DNA) software [16] for GeneChip^® ^Human Mapping 50 K Xba Array, and Genomic Segmentation algorithm of Partek Genomic Suite for Genome-Wide Human SNP Array 6.0. Single nucleotide polymorphisms with smoothing value lower and greater than 2 ± 0.28 were considered as loss and gain, respectively. The profiles were visualized with the VAMP software [17] or Partek Genomic Suite.

### Gene expression data analysis

A series of 89 ovarian primary tumors and 36 ovarian metastases from breast cancer with a clear pathological diagnosis was used to establish a reference hierarchical tree. All these samples were provided by the Resource Biological Center of the Institut Curie and the chips were processed and hybridized in our laboratory (Department of Translational research). The dataset is publicly available on GEO http://www.ncbi.nlm.nih.gov/geo/ under accession number GSE20565. RNAs were prepared according to the manufacturer's instructions, and were hybridized onto Affymetrix GeneChip^® ^Human Genome U133 plus 2.0 Arrays. Transcriptomic data were normalized with gc-Robust Multi-array Average (gcRMA) algorithm [18], using Partek Genomic Suite (Partek Inc., St Louis, MO, USA). Unsupervised hierarchical clustering of tumor samples was done using Partek Genomic Suite software with standard Pearson's correlation as similarity measure, and Ward's method as linkage criteria. The IQR (a measure of the dispersion of each probe set intensity value across all samples) was set in order to have 2 000 probe sets. First, the clustering was performed on this set of reference samples (89 primary tumors and 36 ovarian metastases), then the 16 ovarian samples with ambiguous diagnosis were introduced in the dataset and the clustering was performed.

### Clustering

Validation of the reference hierarchical tree was performed using R environment and the *clusterStab *package [19]. This package assessed the number of reliable clusters and the stability of the hierarchical clustering with a re-sampling approach whereby randomly selected subsets of samples (70% each round) are repeatedly clustered. The extent of similarity between the resulting clusters was examined and measured by the Jaccard coefficient ranging from zero (no similarity) to one (identical clustering). We used this strategy for a number of clusters ranging from 2 to 8, and we compared the results of Jaccard distribution. Enrichment of values equal or close to one indicated adequate choices of metric, agglomeration method and number of clusters. The algorithm was run with the commonly used metrics (Euclidean and Pearson correlation), and the commonly used agglomeration methods (average and Ward's method). The script of the function was adapted in order to use the Pearson correlation coefficient as metric (not implemented in the Bioconductor package). We used the *Hmisc *package from Bioconductor to calculate this correlation between samples.

**Figure 1:**
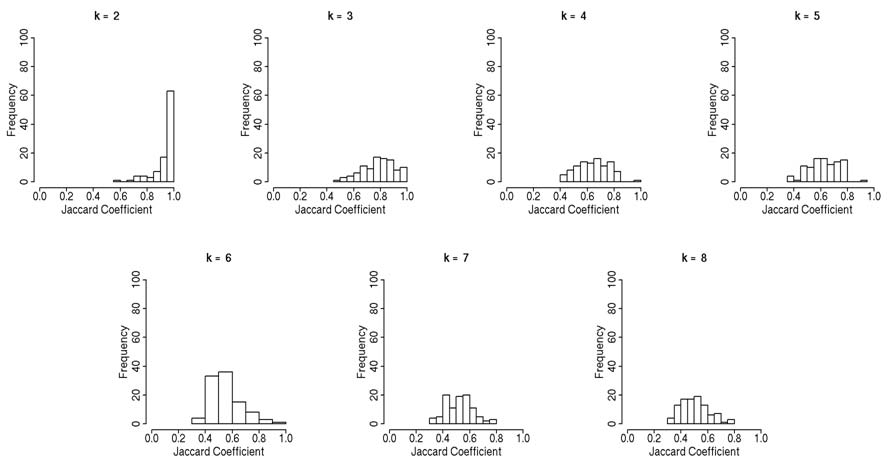
**Distributions of Jaccard coefficients, for a number of clusters ranging from k = 2 to k = 8, derived from 100 independent random samplings of tumors**. Distributions were presented only if Pearson correlation coefficient and Ward's method were used. The proportion of samples used for re-sampling was 0.7. For k = 2 clusters, the largest proportion of values near one indicated that tumors set up two stable clusters.

The use of *clusterStab *package showed that the best reliability of the number of clusters was detected when using Pearson correlation coefficient as metric, Ward's method as agglomeration method, and k = 2 clusters (Figure 1).

## Results

### Patient and tumor characteristics

The main characteristics of the sixteen patients and their tumors are summarized in Table 1. The median age at diagnosis of breast cancer was 48 years (range, 32 to 69 years), and at diagnosis of ovarian tumor was 54 years (range, 38 to 71 years). The median time interval between breast cancer and ovarian tumor diagnosis was 73 months (range, 0 to 150 months). One patient had an ipsilateral breast relapse contemporaneous to the ovarian tumor (case #2). All patients had a breast surgery plus axillary dissection. Only two patients did not receive any systemic therapy. Other patients received a systemic treatment consisting of adjuvant chemotherapy, tamoxifen alone or chemoendocrine therapy according to institutional guidelines. All patients were irradiated according to our institutional guidelines. At a median follow-up of 39 months after the diagnosis of ovarian tumor (range, 1 to 87 months), five patients were still alive, among whom four had no evidence of disease. The median overall survival from breast cancer diagnosis was 78 months, and 29 months from ovarian tumor diagnosis.

**Table 1:** Demographics, treatment, characteristics of breast and ovarian tumors, and disease outcome of the sixteen patients

		Breast tumor				Ovarian tumor				
				
Case #	BRCA status	Age at diagnosis	Stage	Local treatment	Systemic treatment	Age at diagnosis	Time interval from BC (*months*)	FIGO stage	Metastatic site	Outcome
1	ND	45	III	M	CT-T	48	34	IV	Liver	DOD
2	ND	48	III	M	CT	48	73*	IV	Liver	DOD
3	ND	45	III	BCS	CT-T	57	150	IV	Bone, liver	DOD
4	ND	59	I	BCS	No	70	130	IV	Peritoneal carcinomatosis, axillary LN	DOD
5	ND	57	III	M	T	58	20	IIIc	NA	DOD
6	ND	49	III	M	CT-T	52	33	IIIc	NA	DOD
7	BRCA2 m	66	I	BCS	CT	67	19	Ic	NA	NED
8	BRCA1 wt, BRCA2 uk	46	III	BCS	CT-T	46	0	IIa	NA	DOD
9	ND	34	III	M	CT-T	45	133	IV	Peritoneal carcinomatosis, supraclavicular LN, brain	SD
10	ND	47	III	M	T	54	83	IIIc	NA	NED
11	BRCA1 m	69	III	M	CT	71	24	IIa	NA	DOD
12	Familial history ¤	47	III	M	CT-T	54	79	IIIc	NA	DOD
13	ND	50	I	BCS	No	57	87	IIc	NA	NED
14	ND	57	II	BCS	T	64	82	IIc	NA	NED
15	BRCA1 wt, BRCA2 wt	32	III	M	CT	38	74	IV	Pleura	DOD
16	ND	48	II	BCS	CT	49	18	IIIc	NA	DOD

BRCA: BReast CAncer gene, ND = not determined; m = mutation; wt = wild type; uk = unknown; M = mastectomy; BCS = breast-conserving surgery; CT = chemotherapy; T = tamoxifen; BC = breast cancer; LN = lymph nodes; NA = not applicable; DOD = died of disease; NED = no evidence of disease; SD = stable disease.

* Patient who developed simultaneously ipsilateral breast cancer relapse and ovarian tumor.

¤ Her mother had a c.669A > G/Gln147GIN variation in the BRCA2 gene.

The pathological features of breast and ovarian tumors are presented in Table 2. For breast tumors, the expression of HER2 was assessed (data not shown) as negative in five patients, and was not available for the eleven remaining patients. HER2 is a marker of aggressiveness, the overexpression of HER2 gene increases the cellular growth and metastatic potentialities. For cases number 6, 11, 15 and 16, the clinical and pathological analyses were not able to draw a definite conclusion regarding the ovarian tumor diagnosis (Table 3) due to the unspecific clinical presentation (i.e.: ovarian carcinoma) or pathological presentation (poorly differentiated carcinoma which could be compatible with either primary or secondary ovarian tumor). Noteworthy, three of these four patients developed previously a non-lobular breast carcinoma (infiltrating ductal carcinoma in two, undifferentiated in one). No anti-PAX8 staining was observed in primary breast carcinoma (data not shown). For the sixteen ovarian tumors, PAX8 was deemed positive in six cases, and negative in ten cases. Based on clinical and pathological characteristics, two patients were treated as metastatic breast cancer, and two as primary ovarian cancer.

**Table 2:** Pathological features of breast and ovarian tumors

	Histology		Grade		ER		PgR	
				
Case #	Breast	Ovary	Breast	Ovary	Breast	Ovary	Breast	Ovary
1	ILC	Lobular carcinoma	I	ND	+	+	+	ND
2	IDC, then ILC*	Lobular carcinoma	III, then II	ND	+	ND	+	ND
3	ILC	Lobular carcinoma	I	ND	+	+	+	ND
4	ILC	Lobular carcinoma	ND	ND	+	+	+	-
5	ILC	Lobular carcinoma	III	ND	+	ND	+	ND
6	ILC	Lobular carcinoma	II	ND	+	ND	-	ND
7	IDC	Serous papillary	III	III	-	ND	-	ND
8	IDC	Serous papillary	III	II	+	-	+	+
9	IDC	Poorly differentiated ADK	III	ND	+	ND	+	ND
10	IDC and ILC (mixed)	Serous papillary	III	III	+	-	+	-
11	IDC	Poorly differentiated ADK	III	II	-	+	-	+
12	ILC	Lobular carcinoma	III	ND	+	+	+	ND
13	IDC	Serous papillary	II	III	+	ND	+	ND
14	IDC	Clear cell	II	II	+	ND	-	ND
15	IDC	Poorly differentiated ADK	II	ND	+	ND	+	ND
16	Undifferentiated	Serous papillary	III	III	-	+	-	+

ND = not determined; ILC = infiltrating lobular carcinoma; IDC = infiltrating ductal carcinoma; ADK = adenocarcinoma; ER = estrogen receptor; PgR = progesterone receptor.

* Initial breast cancer was an IDC, and local relapse was an ILC (subject of genomic analysis).

**Table 3:** Comparison between pathological analyses, CGH, transcriptomic profiles, and immunohistochemistry profiles

Case #	Pathological analysis	Genomic analysis	Transcriptomic analysis	PAX8 IHC
1	Metastasis	ND	Metastasis	Negative
2	Metastasis	Metastasis	Metastasis	Negative
3	Metastasis	Metastasis	Metastasis	Negative
4	Metastasis	Metastasis	Metastasis	Negative
5	Metastasis	Metastasis	Metastasis	Negative
6	Plausible metastasis	Metastasis	Metastasis	Negative
7	Primary tumor	Primary tumor	Primary tumor	Positive
8	Primary tumor	Primary tumor	Primary tumor	Positive
9	Metastasis	Metastasis	Metastasis	Negative
10	Primary tumor	Primary tumor	Primary tumor	Positive
11	Plausible primary tumor	Primary tumor	Primary tumor	Positive
12	Metastasis	Metastasis	Metastasis	Negative
13	Primary tumor	Primary tumor	Primary tumor	Positive
14	Primary tumor	Primary tumor	ND	Negative
15	Plausible metastasis	Metastasis	Metastasis	Negative
16	Plausible primary tumor	Primary tumor	Primary tumor	Positive

PAX8: Paired Box 8 gene; ND = not determined; IHC = immunohistochemistry.

### SNP array analysis

In four cases out of sixteen ovarian tumors, the pathological diagnosis was uncertain (Table 3). The comparison of mammary and ovarian genomic profiles was first established in the twelve available pairs with a clear diagnosis.

**Figure 2:**
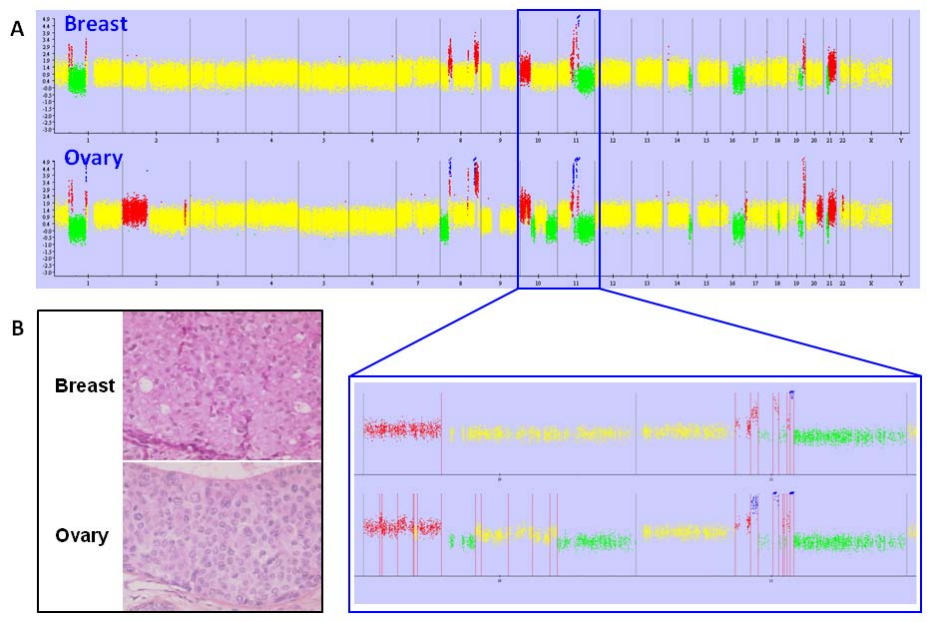
**(A) Genomic profile of the breast/ovary sample pair #9**. In the zoomed area, similar altered areas and breakpoint positions (red vertical lines) between mammary and ovarian tumors were detected. Yellow = normal; red = gain; green = loss; blue = amplification. X axis: all chromosomes, Y axis: SNP copy number. **(B) Histological sections of breast (upper) and ovarian (lower) tumors**

In the subset of seven metastatic samples, one array was not usable for analysis (signal intensity too noisy on the chip). Among the six remaining samples, similar genetic events were found in mammary and ovarian tumors. The alterations found in breast samples were observed in ovarian samples, whereas some additional alterations were observed in the ovarian samples as visually depicted in Figure 2. Moreover, the breakpoints detected in the mammary tumor were also found at the same position in the ovarian sample. Additional breakpoints were also found in this subset of ovarian tumors. These observations led to the conclusions that these six ovarian tumors were metastases of the primary breast cancer.

**Figure 3:**
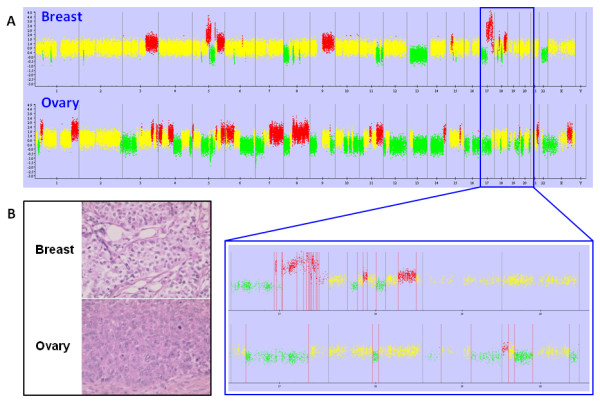
**(A) Genomic profile of the breast/ovary sample pair #8**. In the zoomed area, no similarities for altered areas and breakpoint positions (red vertical lines) between mammary and ovarian tumors were detected. Yellow = normal; red = gain; green = loss; blue = amplification. X axis: all chromosomes, Y axis: SNP copy number. **(B) Histological sections of breast (upper) and ovarian (lower) tumors**

In the other subset of five samples considered as primary ovarian carcinoma, no overlap of genomic alterations was found between mammary and ovarian samples (Figure 3). As well, no common position for breakpoints was observed, thus confirming the primary status of the ovarian tumors (Table 3).

Then, molecular diagnosis was performed on the four ambiguous tumors. Two of them were classified as metastases from breast cancer as similar genetic events were observed in the primary tumor and its ovarian counterpart (see Additional Files 1 and 2). The two remaining tumors were classified as primary tumors as their genetic profiles were strikingly different from those of the original breast tumors (see Table 3 and Additional Files 3 and 4).

### Transcriptomic analysis

**Figure 4:**
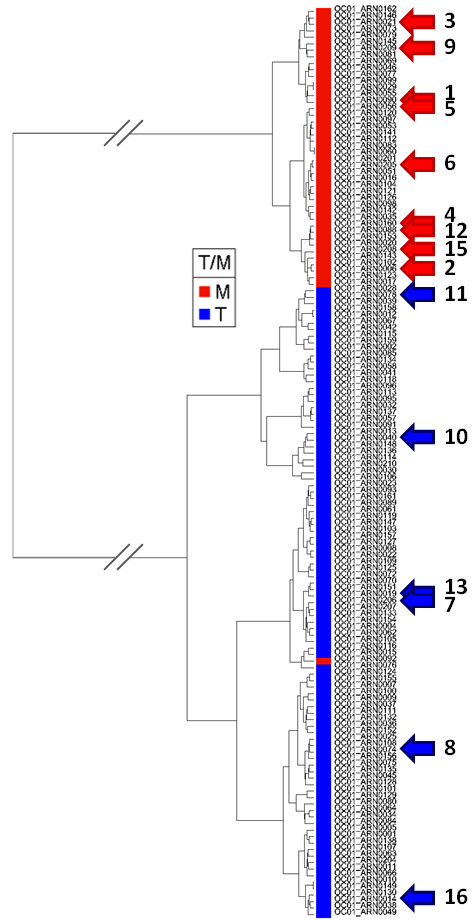
**Hierarchical clustering**. Pearson's correlation coefficient and Ward's method were used. In red are the ovarian metastases samples, and in blue the ovarian primary tumors. Red and blue arrows localized the ovarian metastases and primary tumor samples, respectively, among the 16 cases included in this study

Fifteen out of sixteen gene expression profiles of ovarian tumors were obtained. These tumors were included in a set of samples including well-identified primary ovarian tumors (n = 89), and ovarian metastases (n = 36). All the samples were processed, normalized, and clusterized as described above. Therefore, a hierarchical tree with two main branches was obtained: the first branch gathered all metastatic samples, and the second branch contained all primary tumors but one (Figure 4). The reference hierarchical tree obtained with the 125 reference tumors was not modified when the fifteen studied tumors were added. Two main branches were repeatedly generated with the raising of the tree from 125 to 140 tumors. The fifteen ovarian tumors of the core subset all segregated accordingly to the pathological diagnosis and their genomic profiles (Figure 4, Table 3). The tumor #1, with no SNP array data, clearly segregated with metastatic tumors.

## Discussion

So far, no definitive molecular profiling of ovarian cancer has been published. Molecular characterization is only based on grade, histo-pathological features and some key genes alterations. As well, no high-throughput molecular data on ovarian metastasis have been reported. This study is the first to use a comprehensive genomic analysis to discriminate primary ovarian carcinomas and metastatic ovarian lesions from breast cancer. A similar approach was evaluated to distinguish primary from secondary ovarian tumors after adenocarcinoma of the endometrium [13]. Using a 19 K CGH array, four cases out of nine remained undetermined. In our data set, the pathological status of ovarian lesions was well established in twelve cases. The blind test of these pairs for SNP and transcriptomic analyses was in agreement with the pathological analysis (Table 3). In the four lesions with an ambiguous pathological diagnosis, SNP and transcriptomic analyses permitted to clearly discriminate primary tumor and metastatic disease. This is also the first time that both transcriptomic and genomic analyses were performed, demonstrating a complete overlap of results with these two kinds of analyses.

The purpose of the present work was not to provide a genomic signature, but to give a diagnosis allowing a treatment decision. Conversely to the study of ovarian tumors after adenocarcinoma of the endometrium using low-density 19 k BAC arrays [13], we used high-density 50 K SNP arrays, showing major chromosomal alterations in all tumors. This method allowed interpretation and comparison of the genomic profiles within the fifteen available pairs of breast and ovarian tumors. When most of genomic alterations found in primary breast tumors were included in the ovarian counterpart, a genomic diagnosis of metastases was proposed and then confirmed by the comparison with the histopathological diagnosis. A previous experience of CGH in metastatic breast cancer has been reported, comparing primary tumor with lymph node metastases, distant metastases or local recurrences in sixteen patients [20]. Genomic profiles comparison showed that additional aberrations were detected in the lymph nodes or distant metastases when compared to the primary tumors. These findings suggest that progression from primary breast cancer to metastasis may be associated with the acquisition of further genetic changes. Overall, in the present series, we confirmed these findings and proposed the use of SNP array technology to provide a definite diagnosis in case of equivocal ovarian lesions.

Furthermore, the present study highlighted that the transcriptomic analysis was always in concordance with the pathological diagnosis in the reference set of well-identified 125 tumors, excepted for one case considered as a metastasis, but clustering with primary tumors (Figure 4). Both analyses (genomic and transcriptomic) were possible in fourteen pairs of tumors, and results were always identical (Table 3). Given the robustness of our reference tree, we have therefore established a dataset of reference that is now available for future research and diagnostic purposes. The main interest of this method is to have at one's disposal a test that does not require the primary tumor to give a diagnosis. Indeed, other authors have reported a similar approach in particular with Carcinomas of Unknown Primary (CUP) [21], suggesting that expression data may help in elucidating the histogenetic origin of metastatic tissue. The future directions might be to enrich our dataset of tumors likely to give ovarian metastases, such as gastric or colorectal carcinomas.

The use of WT1 in IHC analysis seems not to be appropriate to discriminate between primary and secondary tumors. A recent study [22] showed that WT1 is rather dedicated to distinguish between serous and endometrioids ovarian carcinomas. So, in our study, we preferred to use PAX8 staining instead of WT1 staining for the distinction of primary and secondary lesions.

Interestingly, PAX8 IHC status was in concordance with genomic results in the present model of primary ovarian tumors compared to ovarian metastases from breast cancer, except for one patient who had a clear-cell carcinoma. PAX8 is usually highly, but not always, expressed in serous-papillary (96,4%), endometrioid (88,9%) or clear-cell carcinoma (100%) [11]. In the series of these authors, PAX8 staining was positive for all the clear-cell carcinoma (10/10), so they concluded that PAX8 was particularly useful for the diagnosis of this histotype. But the only one sample which is not stained in our collection is a clear-cell ovarian carcinoma. Moreover, no definite nor consensual criteria for PAX8 IHC positivity has been defined so far, yielding some concern about the reproducibility of their technique. To our opinion, PAX8 IHC does not provide a totally robust result concerning the status of ovarian tumors. In the present series, genomic analyses yielded a definite diagnosis in all cases. In another study concerning mouse model of human ovarian endometrioid adenocarcinoma [23], the authors performed IHC and sequence analysis, in parallel, on some genes implicated in the pathology. For CTNNB1 gene, the results were totally concordant between IHC and sequence analyses. But for other genes, like PTEN and TP53, there were some discrepancies. For example, 12 samples were found to be mutated with IHC for PTEN whereas when the authors examined the sequence, mutations were found only in 6 samples. In this case, they detected 6 false positives with IHC analysis. Also, when they analyzed TP53 mutations, they detected 3 false positives and 2 false negatives between IHC and sequence analyses. These results demonstrate that genomic tools are more appropriate to determine definitively the mutational status of genes.

## Conclusions

**Figure 5:**
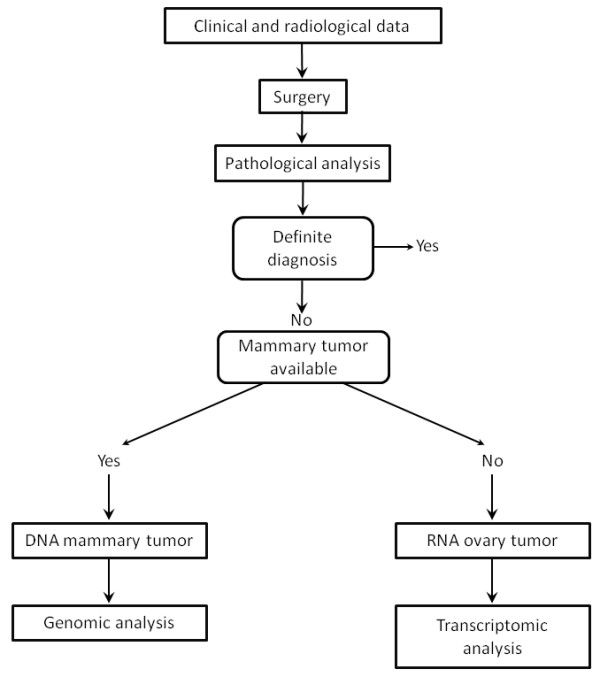
**Proposed diagnostic algorithm of ovarian tumors in patients with a previous history of breast cancer**. If the mammary tumor of the patient is available, it is possible to perform a comparison between genomic profile of breast and ovary. Otherwise, if only the ovary tumor is available, a transcriptomic analysis is performed, and the sample is inserted into the reference hierarchical tree.

We established the robustness of SNP and transcriptomic analysis to discriminate primary ovarian tumors and ovarian metastases after primary breast cancer. Noteworthy, all genomic analyses were blindly compared to clinical and pathological characteristics. An algorithm of diagnosis could be considered in the light of these results (Figure 5). The first step remains the pathological analysis. If the primary tumor is not available, which represents the majority of cases, a transcriptomic analysis may be performed. If the primary tumor is available, the SNP array is the preferential test. Of course, these results require further analyses and comparison with other biomarkers.

## Abbreviations

ADK: Adenocarcinoma; AJCC: American Joint Committee on Cancer; BCS: Breast-Conserving Surgery; BC: Breast Cancer; BRCA-1(-2): BReast CAncer-1(-2) gene; CA-125: Carbohydrate Antigen 125; CA 15-3: Carbohydrate Antigen 15-3; CGH: Comparative Genomic Hybridization; CT: ChemoTherapy; CT-scan: Computed Tomography-scan; DNA: DesoxyriboNucleic Acid; DOD: Died Of Disease; EDTA: Ethylene Diamine Tetraacetic; ER: Estrogen Receptor; FIGO: Fédération Internationale de Gynécologie Obstétrique; gcRMA: gc-Robust Multi-array Average; GLAD: Gain and Loss Analysis of DNA; HER2: Human Epidermal Growth Factor Receptor-2; IDC: Infiltrating Ductal Carcinoma; IHC: ImmunoHistoChemistry; ILC: Infiltrating Lobular Carcinoma; IQR: InterQuartile Range; ITALICS: ITerative and Alternative normaLIzation and Copy number calling for affymetrix Snp arrays; LN: Lymph Nodes; m: Mutation; M: Mastectomy; NA: Not Applicable; ND: Not Determined; NED: No Evidence of Disease; PAX8: Paired Box 8 gene; PBS: Phosphate Buffered Saline; PgR: Progesterone Receptor; RIN: RNA Integrity Number; RNA: Ribonucleic Acid; SD: Stable Disease; SNP: Single Nucleotide Polymorphism; T: Tamoxifen; UICC: Union Internationale Contre le Cancer; uk: Unknown; VAMP: Visualization and analysis of Array-CGH, transcriptome and other Molecular Profiles; WT1: Wilms Tumor suppressor gene; WT: Wild Type

## Competing interests

The authors declare that they have no competing interests.

## Authors' contributions

JPM, with the support of CD, AR, DG and SRR, conceived of the study, participated in its design and coordination, carried out the translational and statistical analysis, and helped to draft the manuscript. PHC conceived of the study, participated in its design and coordination and helped to draft the manuscript. PHC and LM carried out the follow-up of patients and reviewed clinical data. JC, IL, AN, NW and XSG carried out the pathologic analysis. VF and SA carried out the surgical procedures. All authors read and approved the final manuscript.

## Pre-publication history

The pre-publication history for this paper can be accessed here:

http://www.biomedcentral.com/1471-2407/10/222/prepub

## Supplementary Material

Additional file 1 **Genomic profile of the breast/ovary sample pair #6 with uncertain diagnosis**. The genomic profiles of this pair were performed using Affymetrix GeneChip^® ^Mapping 50 K Xba Array. The normalization and segmentation methods used for this kind of array were those described in Materials and Methods. Few alterations were detected in the breast and the ovary samples. In the zoomed area, we see that an alteration and breakpoints (red vertical lines) are detected at the same positions in the 2 samples, indicating that the ovary tumor is a metastasis from the breast. Yellow = normal; red = gain; green = loss; blue = amplification. X axis: all chromosomes, Y axis: SNP copy number. Click here for file

Additional file 2 **Genomic profile of the breast/ovary sample pair #15 with uncertain diagnosis**. The genomic profiles of this pair were performed using Affymetrix GeneChip^® ^Mapping 50 K Xba Array. The normalization and segmentation methods used for this kind of array were those described in Materials and Methods. In the zoomed area, we observe that common alterations are found between the breast and the ovary tumors. Moreover, breakpoints (red vertical lines) are detected exactly at the same positions between the 2 samples. So, in that case, the ovary tumor is a metastasis from the breast. Yellow = normal; red = gain; green = loss; blue = amplification. X axis: all chromosomes, Y axis: SNP copy number. Click here for file

Additional file 3 **Genomic profile of the breast/ovary sample pair #11 with uncertain diagnosis**. The genomic profiles of this pair were performed using Affymetrix Genome-Wide Human SNP Array 6.0 Array. The normalization and segmentation methods used for this kind of array were those described in Materials and Methods. Only the first 6 chromosomes are shown but they are representatives of all the alteration profiles observed on the 2 samples. In each chromosome graph, the 2 top profiles represent the chromosomal copy number, the 2 bottom graphs represent the result of genomic segmentation algorithm: red area = gain, blue area = loss. We can observe that no common alterations are detected between the breast and the ovary samples, indicating that the breast and the ovary tumors are both primary tumors. Click here for file

Additional file 4 **Genomic profile of the breast/ovary sample pair #16 with uncertain diagnosis**. The genomic profiles of this pair were performed using Affymetrix GeneChip^® ^Mapping 50 K Xba Array. The normalization and segmentation methods used for this kind of array were those described in Materials and Methods. Along the chromosomes and in the zoomed area, no common alteration or breakpoint (red vertical lines) position is detected between the breast and the ovary tumors. This result reveals that the ovary tumor is a primary tumor and not a metastasis from the breast. Yellow = normal; red = gain; green = loss; blue = amplification. X axis: all chromosomes, Y axis: SNP copy number. Click here for file
