# Supplementary figures and images for: A genomic and transcriptomic approach for a differential diagnosis between primary and secondary ovarian carcinomas in patients with a previous history of breast cancer
Source: BMC Cancer. 2010 May 21;10:222. doi: 10.1186/1471-2407-10-222 (PMC2891634; doi:10.1186/1471-2407-10-222)

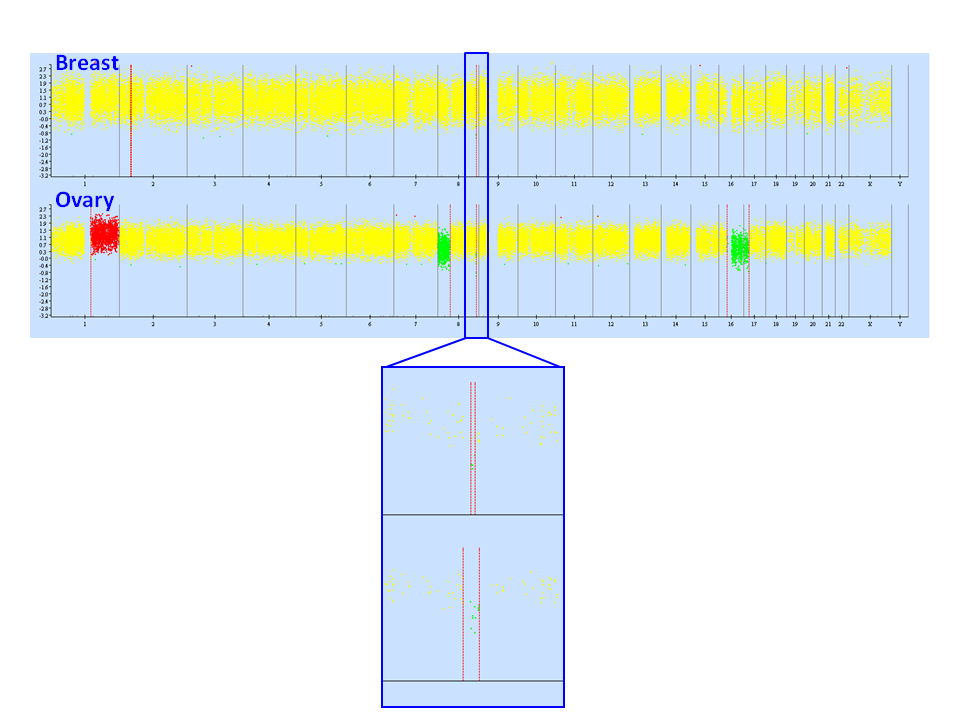

Supplement: Additional file 1 — Genomic profile of the breast/ovary sample pair #6 with uncertain diagnosis. The genomic profiles of this pair were performed using Affymetrix GeneChip® Mapping 50 K Xba Array. The normalization and segmentation methods used for this kind of array were those described in Materials and Methods. Few alterations were detected in the breast and the ovary samples. In the zoomed area, we see that an alteration and breakpoints (red vertical lines) are detected at the same positions in the 2 samples, indicating that the ovary tumor is a metastasis from the breast. Yellow = normal; red = gain; green = loss; blue = amplification. X axis: all chromosomes, Y axis: SNP copy number. [file 1471-2407-10-222-S1.TIFF]

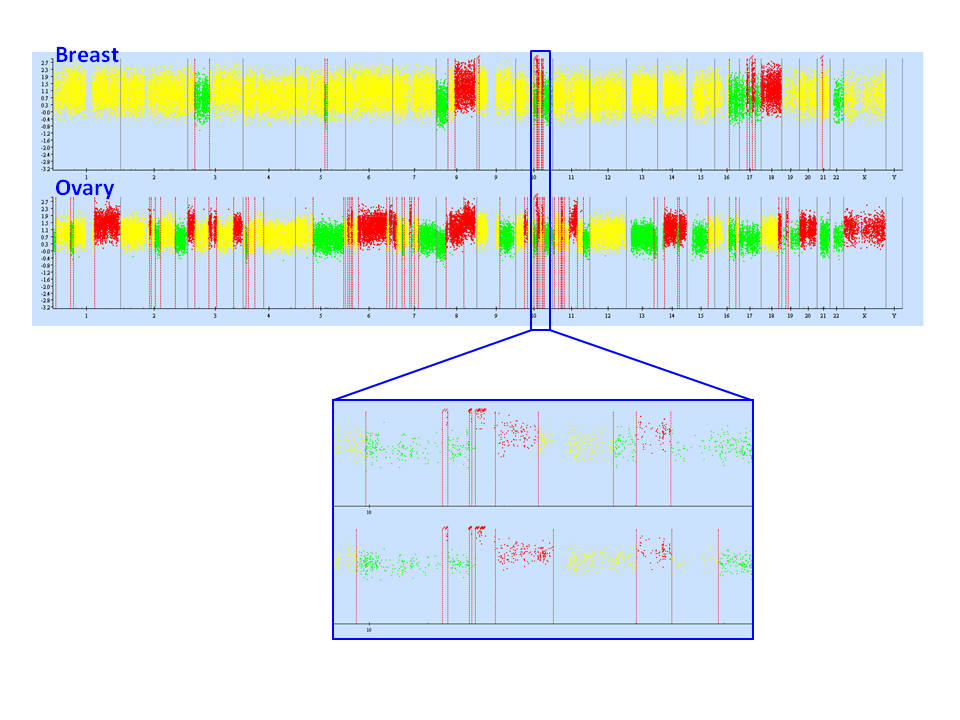

Supplement: Additional file 2 — Genomic profile of the breast/ovary sample pair #15 with uncertain diagnosis. The genomic profiles of this pair were performed using Affymetrix GeneChip® Mapping 50 K Xba Array. The normalization and segmentation methods used for this kind of array were those described in Materials and Methods. In the zoomed area, we observe that common alterations are found between the breast and the ovary tumors. Moreover, breakpoints (red vertical lines) are detected exactly at the same positions between the 2 samples. So, in that case, the ovary tumor is a metastasis from the breast. Yellow = normal; red = gain; green = loss; blue = amplification. X axis: all chromosomes, Y axis: SNP copy number. [file 1471-2407-10-222-S2.TIFF]

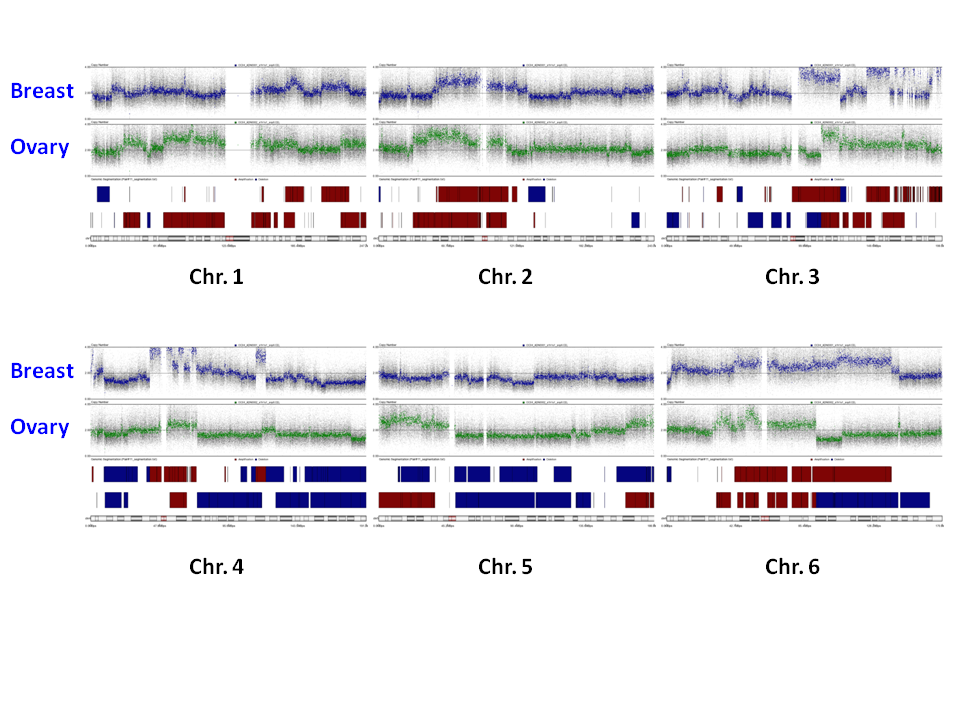

Supplement: Additional file 3 — Genomic profile of the breast/ovary sample pair #11 with uncertain diagnosis. The genomic profiles of this pair were performed using Affymetrix Genome-Wide Human SNP Array 6.0 Array. The normalization and segmentation methods used for this kind of array were those described in Materials and Methods. Only the first 6 chromosomes are shown but they are representatives of all the alteration profiles observed on the 2 samples. In each chromosome graph, the 2 top profiles represent the chromosomal copy number, the 2 bottom graphs represent the result of genomic segmentation algorithm: red area = gain, blue area = loss. We can observe that no common alterations are detected between the breast and the ovary samples, indicating that the breast and the ovary tumors are both primary tumors. [file 1471-2407-10-222-S3.TIFF]

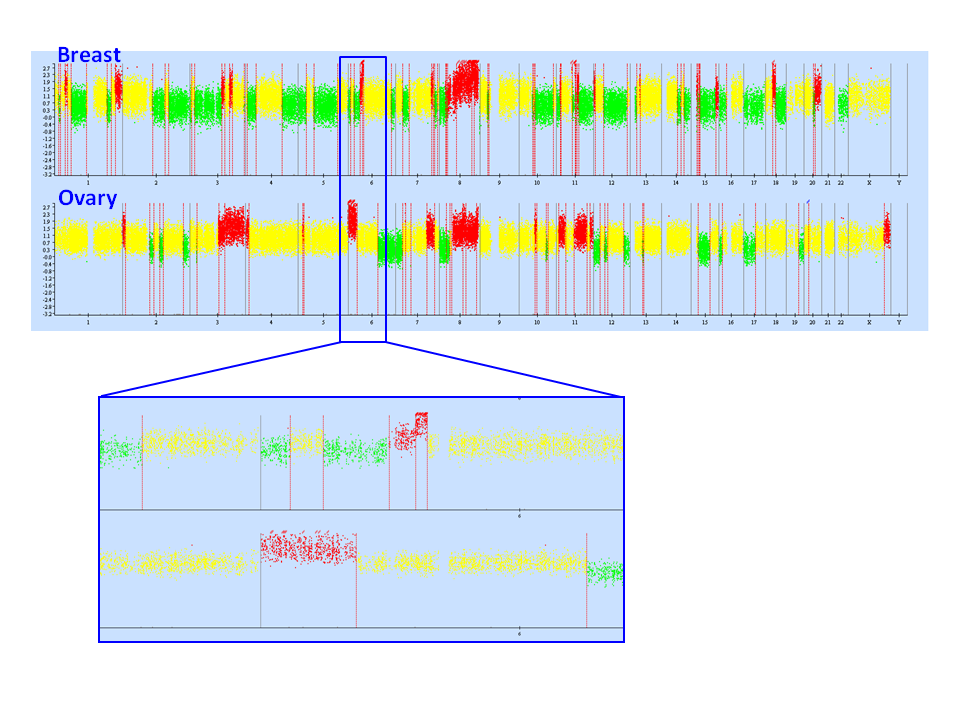

Supplement: Additional file 4 — Genomic profile of the breast/ovary sample pair #16 with uncertain diagnosis. The genomic profiles of this pair were performed using Affymetrix GeneChip® Mapping 50 K Xba Array. The normalization and segmentation methods used for this kind of array were those described in Materials and Methods. Along the chromosomes and in the zoomed area, no common alteration or breakpoint (red vertical lines) position is detected between the breast and the ovary tumors. This result reveals that the ovary tumor is a primary tumor and not a metastasis from the breast. Yellow = normal; red = gain; green = loss; blue = amplification. X axis: all chromosomes, Y axis: SNP copy number. [file 1471-2407-10-222-S4.TIFF]
